
JOURNAL INFORMATION
==============================
NLM Title Abbreviation: ScientificWorldJournal
Journal ID: ScientificWorldJournal
NLM Journal ID: 101131163
Journal ID: TSWJ

The Scientific World Journal

ISSN: 2356-6140
EISSN: 1537-744X
Publisher: Wiley

ARTICLE INFORMATION
==============================
PMCID: PMC6084200
DOI: 10.1100/tsw.2001.449
Article ID: 856864
Article version: 1
Subjects: Extended Abstract

Amylin as a Neuroendocrine Hormone

Young Andrew A.
Vice President Research, Amylin Pharmaceuticals Inc, 9373 Towne Centre Drive, San Diego, CA 92121, USA
Publication date: 2001
Electronic publication date: 2001 Nov 23
Volume: 1
First page: 24
Last page: 24
Copyright: Copyright © 2001 Andrew A. Young.
Copyright year: 2001
License: This is an open access article distributed under the Creative Commons Attribution License, which permits unrestricted use, distribution, and reproduction in any medium, provided the original work is properly cited.
License URL: https://creativecommons.org/licenses/by/3.0/

==============================
4th International Meeting on Calcitonin Gene-Related Peptide (CGRP)
*TheScientificWorld* (2001) 1(S1), 24
ISSN 1532-2246; DOI 10.1100/tsw.2001.449

### AMYLIN AS A NEUROENDOCRINE HORMONE

Andrew A. Young
Vice President Research, Amylin Pharmaceuticals Inc, 9373 Towne Centre Drive, San Diego, CA 92121, USA. Ph (619) 642-7122, FAX (619) 552-2212
E-mail ayoung@amylin.com

Amylin is released with insulin from pancreatic β-cells in response to nutrients and other insulinogenic stimuli. Amylin's most potent actions include slowing of gastric emptying, suppression of postprandial glucagon secretion, reduction of food intake, and inhibition of secretion of acid and digestive enzymes from the stomach and exocrine pancreas, respectively. These actions point to a physiologic function of amylin to regulate the rate at which nutrients are assimilated and released into the circulation. Effects of replacement therapy in patients with absolute amylin deficiency (type 1 diabetes) or relative amylin deficiency (late type 2 diabetes) using the human amylin analog, pramlintide, include improved glycemic control and reduced body weight, consistent with such a physiologic function.

Several, if not all, of amylin's actions appear to be centrally mediated. Focal lesioning studies indicate that effects upon gastric emptying and food intake at least, involve the *area postrema*, a circumventricular organ devoid of a blood-brain-barrier that has access to circulating peptides. Some actions (slowing of gastric emptying) require an intact vagus nerve, while others (postprandial glucagonostatic effect, effect on food intake) do not. Several amylinergic actions (slowing of gastric emptying, inhibition of nutrient-stimulated glucagon secretion, inhibition of gastric acid secretion) are glucose-dependent, in that they are over-ridden during hypoglycemia. The glucose-dependence of some amylin actions may be explained by the finding that the activity of almost all amylin-sensitive neurones in single-unit extracellular recordings of *area postrema* were glucose-sensitive, showing inhibition by the presence of a low ambient glucose concentration.

Amylin binds at picomolar concentrations to several brain regions, including circumventricular organs (*area postrema*; subfornical organ) and *nucleus accumbens*. A distinctive amylinergic pharmacology has been identified in preparations from those sites by selective agonists and antagonists. This pharmacology is replicated in amylin receptors reconstituted as the heterodimer formed by coexpression of the calcitonin receptor with receptor activity modifying protein 1 (RAMP1).

In summary, via several parallel centrally-mediated glucose-dependent actions, amylin physiologically regulates the rate of entry of nutrient into the circulation. Its function may thus be viewed as complementing that of insulin (secreted from the same pancreatic β-cells), which controls the exit of nutrient from blood and promotes nutrient storage in peripheral tissues.
